# Double-mode and double-beam staggered double-vane traveling-wave tube with high-power and broadband at terahertz band

**DOI:** 10.1038/s41598-022-15975-0

**Published:** 2022-07-14

**Authors:** Wenbo Wang, Zheng Zhang, Pengpeng Wang, Yaqi Zhao, Feng Zhang, Cunjun Ruan

**Affiliations:** 1 School of Electronics and Information Engineering, Beihang University, Beijing, 100191 China; 2 Beijing Key Laboratory for Microwave Sensing and Security Application, Beihang University, Beijing, 100191 China

**Keywords:** Electrical and electronic engineering, Design, synthesis and processing

## Abstract

In this paper, a 220 GHz broadband and high-power staggered double-vane traveling-wave tube has been designed and verified. Firstly, a planar double-beam staggered double-vane slow-wave structure is proposed. By using the double-mode operation scheme, the transmission performance and bandwidth have been almost increased with twice as the single mode. Second, to satisfy the high output power requirement and improve the stability of the traveling-wave tube, a double pencil-beam electron optical system has been designed, the 20–21 kV driven voltage and the 2 × 80 mA current are set as the design target. By using the mask portion and the control electrode in the double beam gun, the two pencil beams can be focused with a compression ratio of 7 along their respective centers with narrow distances of about 0.18 mm with good stability. The uniform magnetic focusing system has also been optimized. The stable transmission distance of planar double electron beams could reach 45 mm with the focusing magnetic field of 0.6 T, which was long enough to cover the whole high-frequency system (HFS). Then, to verify the availability of the electron optical system and the performance of the slow-wave structure, particle-in-cell (PIC) simulation has also been carried out with the whole HFS. Results demonstrate that the beam-wave interaction system can get a nearly 310 W peak output power at 220 GHz with a 20.6 kV optimized beam voltage and beam current of 2 × 80 mA, the gain is of 38 dB with the 3-dB bandwidth over 35 dB about 70 GHz. Finally, the high precision microstructures fabrication has been carried out to verify the performance of the HFS, the results show that the bandwidth and the transmission properties are in good agreement with simulation result. Thus, the proposed scheme in this paper is expected to develop the high-power and ultra-wideband terahertz band radiation source with potential applications in the future.

## Introduction

As a traditional vacuum electron device, traveling-wave tube (TWT) plays an irreplaceable role in many applications like high-resolution radar, satellite communication systems, space exploration, and so on^1–3^. However, as the working frequency enters the terahertz band, the traditional coupled cavity TWT and helix TWT cannot meet the needs of people due to their relatively low output power, narrow bandwidth and difficult fabricating process. Thus, how to comprehensively improve the performance of TWT at terahertz band has become a very important question for many scientific research institutions. Recently, some new Slow-Wave Structures (SWSs) such as staggered double-vane (SDV) structure and folded-waveguide (FW) structure have attracted wide attention due to their natural planar structure, especially the novelty SDV-SWS with good potential. This structure was proposed by UC-Davis in 2008^4^. The planar structures can be easily fabricated by using the micro/nano machining technologies like Computer Numerical Control (CNC) and UV-LIGA, the all metal-package structure can provide a large thermal capacity with a higher output-power and gain, and the waveguide-like structure can also provide a wider operating bandwidth. At present, SDV-TWTs have firstly been proved to produce over 100-W high-power outputs and nearly 14 GHz bandwidth signal at G-band in 2017 by UC Davis^5^. However, these results still exist gaps and cannot meet the relevant requirements of high power and wide bandwidth at terahertz band. For the G-band SDV-TWT of UC-Davis, the sheet electron beam has been used. Although the scheme can significantly promote the beam’s current capacity, it is difficult to realize the sheet beam electron optical system (EOS) to maintain long transmission distance because of the instability, and there is an over-mode beam tunnel which may also cause beam self-excitation and oscillation^6,7^. In order to achieve the requirements of high output power and broad bandwidth with good stability for terahertz TWT, a double-beam SDV-SWS with double-mode operation has been proposed in this paper. That is to say, to enhance the working bandwidth, the double-mode operation has been proposed and introduced in this structure. And, to improve the output power, the planar distributed double pencil beams are also used. Because of the limitation of the vertical size, the radio of the single pencil beam is relatively small. In case the current density is too high, the current of the beam must be reduced, which results in the relatively low output power. In order to improve the beam current, planar distributed multi-beam EOS has appeared, which uses the lateral size of the SWS. Due to the independent beam tunnels, planar distributed multi-beam can achieve high output power by sustaining the total high beam current with a small current for each beam, which may avoid the over-mode beam tunnels compared with the sheet beam devices. Thus, it is beneficial to keep the stability of the TWT. Based on the previous work^8,9^, a uniform magnetic field focusing double pencil-beam EOS at G-band has been proposed in this paper, which can greatly improve the stability transmission distance of the beam and further increase the beam-wave interaction area, thus greatly improving the output power.

The structure of this paper is as follows. First, the unit cell design of the SWS with parameters, dispersion characteristic analyses and high-frequency simulation results have been described. Then, according to the structure of the unit cell, both the double pencil-beam EOS and the beam-wave interaction system have been designed in this paper. Particle-in-cell simulation results have also been demonstrated to verify the availability of the EOS and the performance of the SDV-TWT. In addition, a brief introduction of the fabrication and cold test results have also been given in this paper to verify the correctness of the whole HFS. And finally makes a summary.

## High-frequency system

### Unit cell design of SDV slow-wave circuit

**Figure 1:**
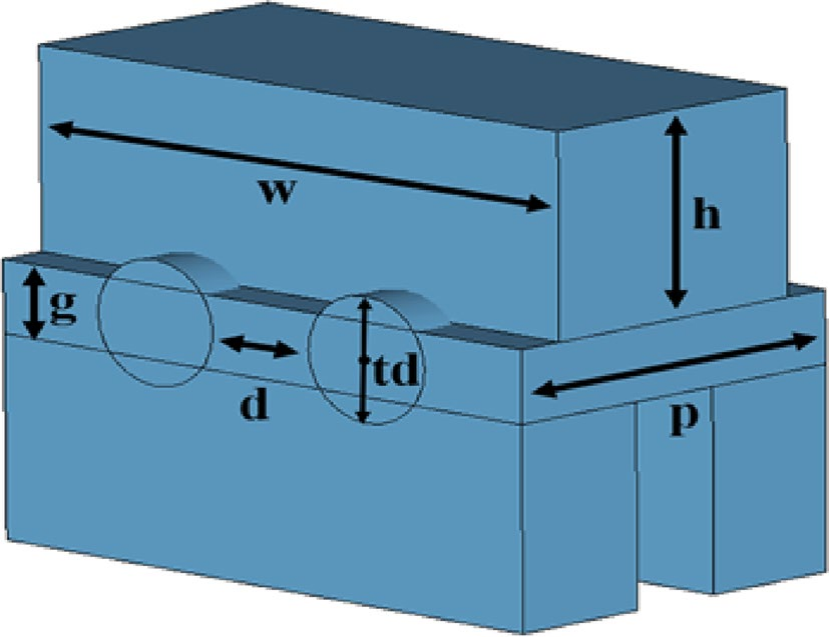
3-D schematic of a unit cell of multi-beam SDV-SWS. The blue section is vacuum.

As one of the most important components of TWT, the dispersion characteristic of the slow-wave structure shows whether the electron velocity matches the phase velocity of the SWS, so, it has a great influence in beam-wave interaction. In order to improve the performance of the whole TWT, an improved interaction structure has been designed. The structure of the unit cell is shown in Fig. 1. Considering the instability of the sheet-beam and the power limitation of the single pencil beam, double pencil beams have been used to the structure to further improve the output power and the operation stability. At the same time, in order to increase the working bandwidth, double-mode operation has been proposed to SWS. Due to the symmetry of SDV structure, solutions of the dispersion equation of electromagnetic field can be divided into odd mode and even mode. The wideband synchronous of beam-wave interaction can be realized by using both the fundamental odd mode in the low-frequency band and the fundamental even mode in the high-frequency band, hence the working bandwidth can be further increased.

According to the power requirement, driven voltage of 20 kV and double-beam current of 2 × 80 mA are used in the design of the whole tube. In order to make the voltage match with the working bandwidth of the SDV-SWS as much as possible, we need to calculate the length of the period ***p***. The relationship between electron-beam voltage and period is shown in Equivalent (1)^10^:

1 $$\begin{array}{c} {\varvec{\varphi}} =\frac{{2\uppi}{\varvec{f}}_{0}{\varvec{p}}}{{\text{c}}\sqrt{\text{1} - \frac{1}{{\left(\text{1} + {\varvec{U}}_{0}/{511}\right)}^{2}}}}\end{array}$$

**Figure 2:**
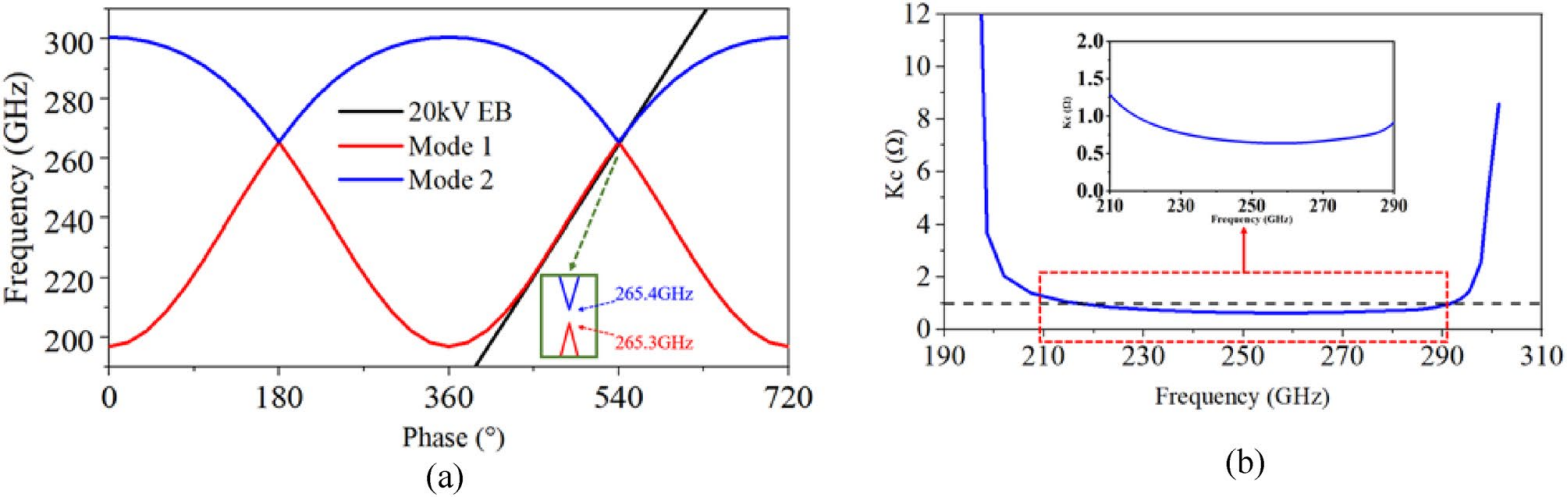
(**a**) Dispersion characteristic of double-mode SDV-SWSs with 20 kV electron beam line. (**b**) Interaction impedance of SDV slow-wave circuit.

By setting the phase shift as 2.5π at the center frequency of 220 GHz, the period ***p*** can be calculated as 0.46 mm. Figure 2a shows the dispersion characteristics of the unit cell of SWS. The 20 kV beam line is well-matched with the double-mode curve. The matching band can reach about 70 GHz in the range of 210–265.3 GHz (odd mode) and 265.4–280 GHz (even mode). Figure 2b shows the average coupling impedance, which is greater than 0.6 Ω from 210 to 290 GHz, this result demonstrates that strong interaction can occur in the operating bandwidth.

However, it should be noted that there is a band gap between odd mode and even mode, and we usually call this band gap as stopband, as shown in Fig. 2a. If the TWT works around this band, a strong beam-wave coupling strength may occur, which will result in unwanted oscillation. In practical application, we usually avoid using TWT around the stopband. However, it can be seen that the band gap of this slow-wave structure is only 0.1 GHz. It’s difficult to confirm whether this small band gap will result in the oscillation. So, in this paper, working stability around the stopband will be studied in the following PIC simulation part to analyze whether the unwanted oscillation will happen.

### Transmission performance of HFS

**Figure 3:**
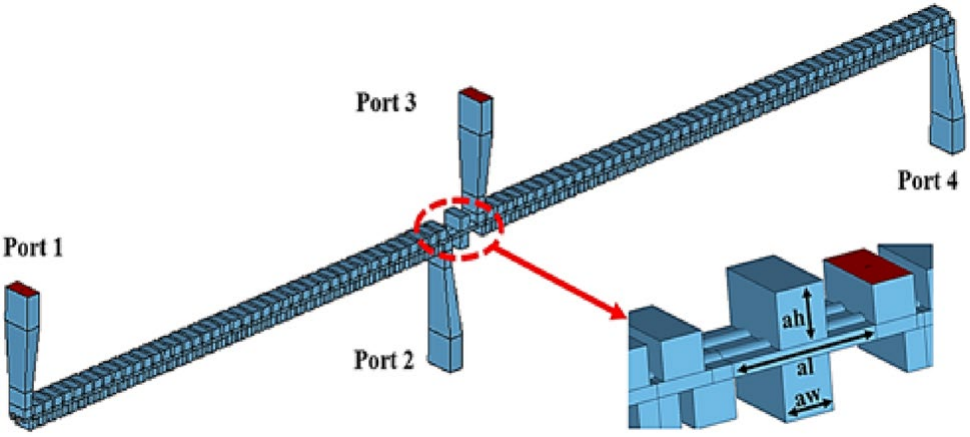
Schematic of two-stage multi-beam slow-wave circuits with couplers and Bragg attenuators.

The model of the whole HFS is shown in Fig. 3. It consists of two stages of SDV-SWS which are connected by Bragg reflector. The function of the reflector is to cut off the transmission of signals between the two stages and suppress the oscillation and reflection of non-working modes such as high-order modes generated from the space between the upper and lower vanes^11^, thus greatly enhancing the stability of the whole tube. In order to connect with the external environment, the linear taper coupler is also used to connect the SWS with the WR-4 standard waveguide. The transmission coefficient of the two-stage structure is measured by time-domain solver in 3D simulation software. Considering the actual influence of the terahertz band on the material, the material of the vacuum envelope is set as copper preliminary and the conductivity is reduced to 2.25 × 10^7^ S/m^12^.

**Figure 4:**
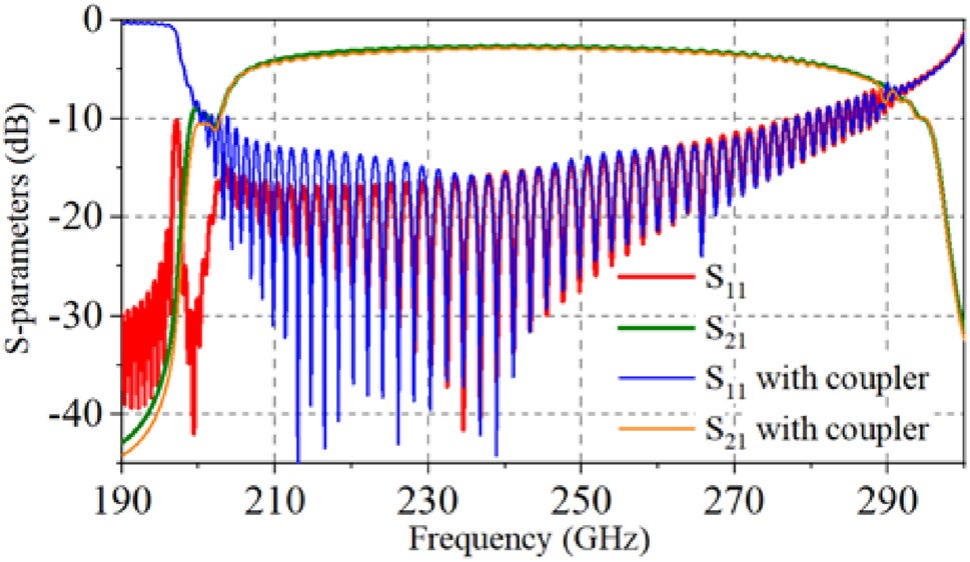
Transmission performances of the slow-wave circuit with and without couplers.

Figure 4 shows the transmission results of the HFS with and without linear taper couplers. The result demonstrates that the coupler has little influence on the transmission performance of the whole HFS. The return loss (S_11_ < − 10 dB) and the insertion loss (S_21_ > − 5 dB) of the whole system in a broad band from 207 to 280 GHz show that the HFS has good transmission characteristics.

## Double beams electron optics system

### Design, optimization and voltage sensitivity analysis of electron gun

As the power source of vacuum electronic device, the electron gun directly determines whether the device can produce enough output power. Combined with the analysis of the HFS in section II, double-beam EOS is needed to be designed which to provide enough electric energy. In this part, a double pencil-beam electron gun is designed by using planar mask portions and control electrodes according to the previous work at W-band^8,9^. First of all, according to the design requirements of the SWS in Sect. 2, the driven voltage ***U***_***a***_ of the electron beam is initially set as 20 kV, both current ***I*** of the two electron beams are 80 mA, and the beam diameter ***d***_***w***_ of the electron beam is 0.13 mm. At the same time, in order to ensure that the current density of electron beam and cathode can be realized, the compression ratio of the electron beam is set to 7, so the current density of the electron beam is 603 A/cm^2^, while the current density at the cathode is 86 A/cm^2^, which can be achieved by using the new cathode materials^13^. According to design theory^14–17^, a typical Pearce electron gun can be uniquely determined.

**Figure 5:**
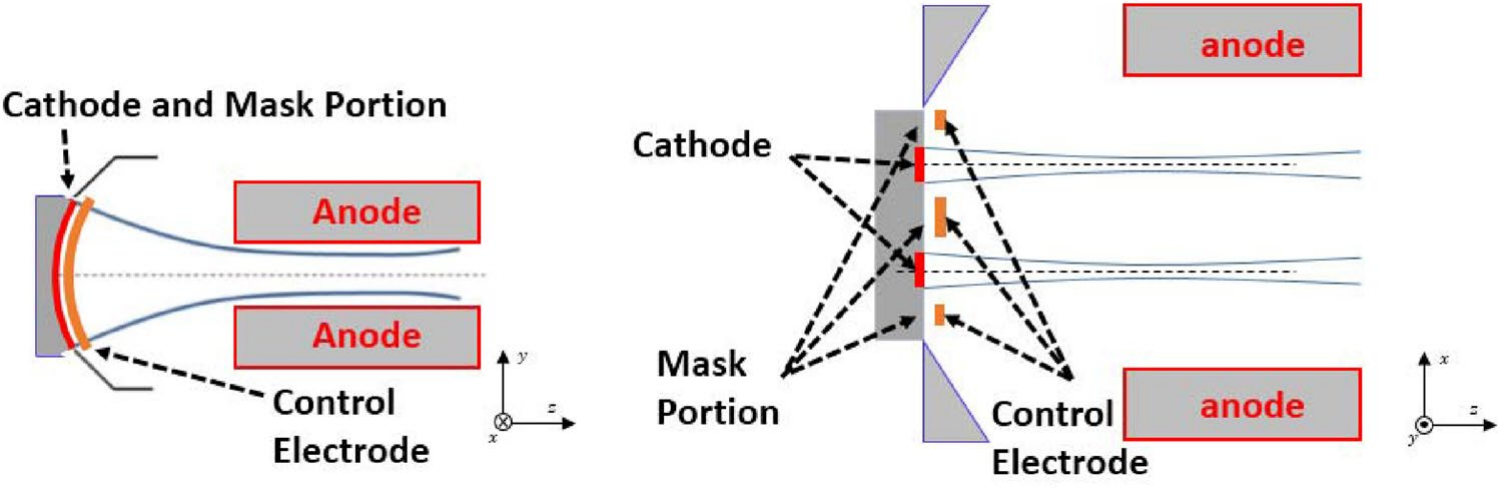
Schematic of the planar double-beam electron gun in both two transverse directions.

Figure 5 shows the schematic diagram of the gun in the transverse and longitudinal directions respectively. It can be seen that the profile of the electron gun in the x-direction is almost the same as that of a typical sheet beam gun, while in the y-direction the two beams are separated by the mask portion. The positions of the two cathodes are at x = − 0.155 mm, y = 0 mm and x = 0.155 mm, y = 0 mm, respectively. According to the design requirements of compression ratio and electron injection size, the size of the two cathode surfaces is determined to be 0.91 mm × 0.13 mm.

**Figure 6:**
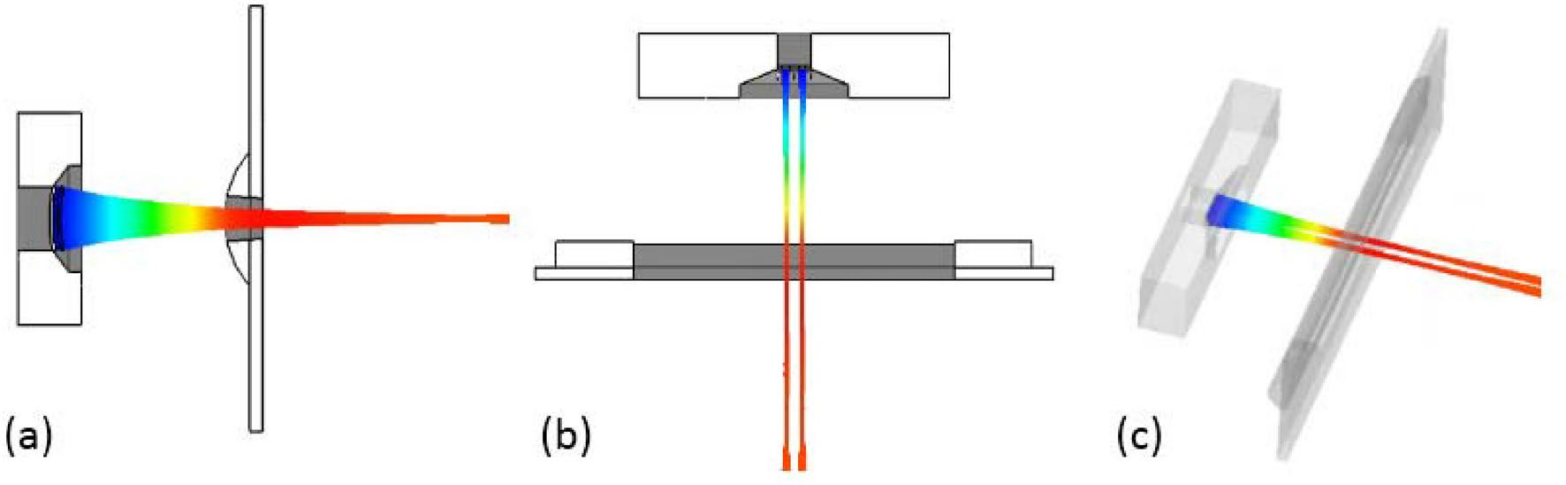
Beam trajectory of the double-beam gun. (**a**) y-o-z plane. (**b**) x-o-z plane. (**c**) 3D view.

In order to make the focused electric field received by each electron beam in the x-direction symmetric along its own center, the control electrode is applied to the gun in this paper. By setting the voltage of − 20 kV at the focusing electrode and the control electrode and 0 V at the anode, we can get the trajectory distribution of the double-beam gun, as shown in Fig. 6. It can be seen that the emitted electrons have good compression in the y-direction, and each beam converges in the x-direction along its own symmetric center, which indicates that the control electrode has balanced the unequal electric field generated by the focusing electrode.

**Figure 7:**
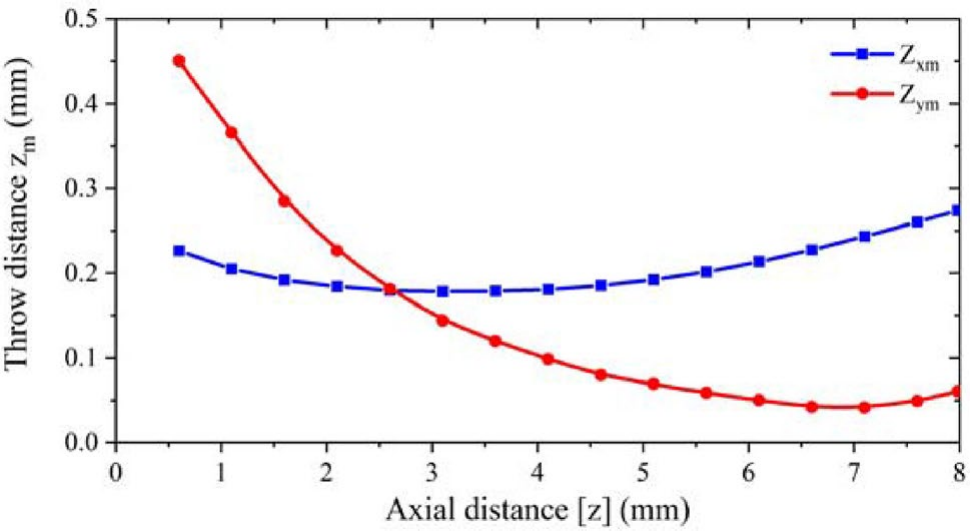
Beam envelopes of the double-beam gun in x and y directions.

**Figure 8:**
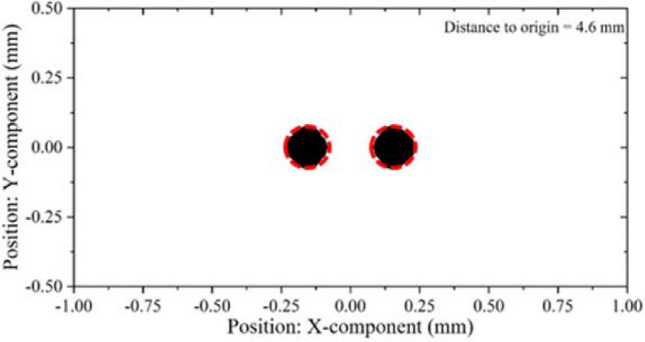
Cross sections of the electron beam at 4.6 mm away from the cathode (without focusing system).

**Figure 9:**
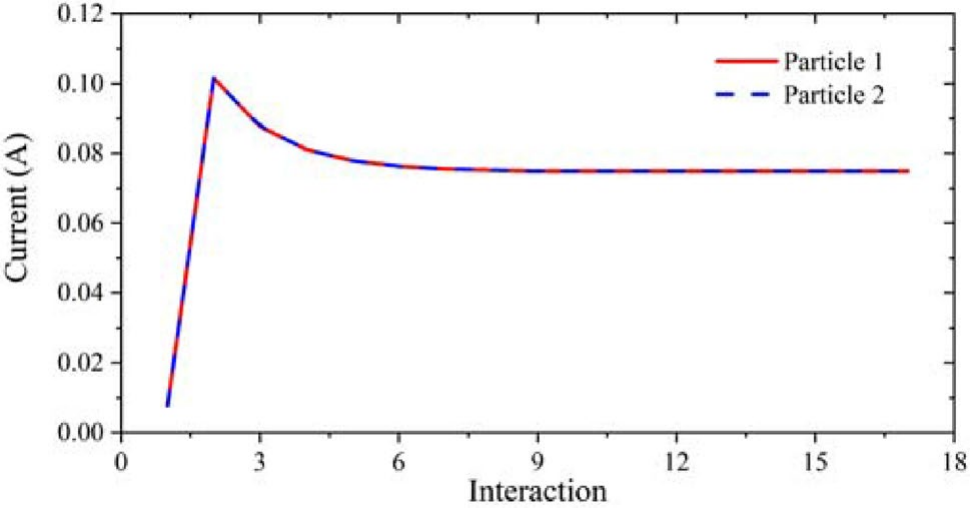
Simulation results of current generated by double-beam electron gun.

Figure 7 shows the beam envelope in x and y directions. The results show that the throw distance of the electron beam in x-direction is different from that in y-direction. The throw distance in x-direction is about 4 mm, while in y-direction is nearly 7 mm. Therefore, the actual throw distance should be selected in a position between 4 and 7 mm. Figure 8 shows the cross sections of the electron beam which is 4.6 mm away from the cathode surface. We can see that the shape of the cross section is closest to the standard circular electron beam. The distance between the two electronic beams is close to the designed 0.31 mm, and the radius is about 0.13 mm, which meets the design requirements. Figure 9 shows the simulation results of beam current. It can be seen that the current of the two beams is 76 mA, which is in good agreement with the designed 80 mA.

**Figure 10:**
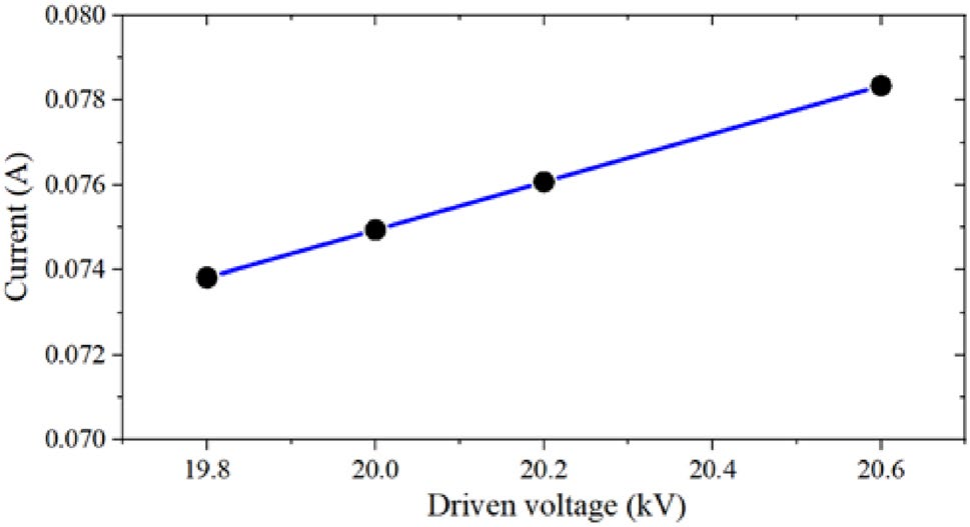
Influence of driven voltage fluctuations on beam current diagram.

**Figure 11:**
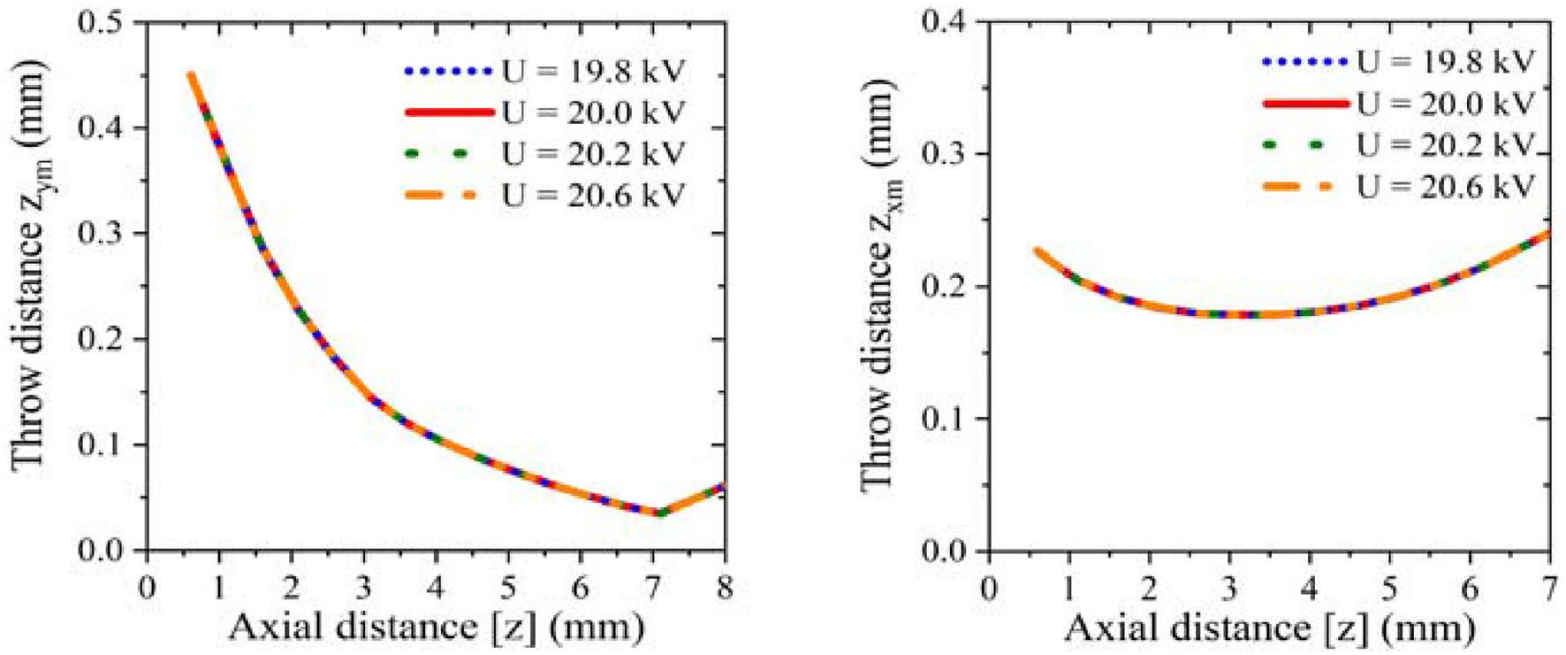
Effects of driven voltage fluctuations on beam envelope diagram in x-direction and y-direction respectively.

Considering the fluctuations of driven voltage in practical applications, it is necessary to research the voltage sensitivity of this model. With the voltage range from 19.8 to 20.6 kV, the current and the beam envelope diagram were obtained, as shown in Figs. 10 and 11. From the results, we can see that the change of the driven voltage has no influence on the beam envelope, and the beam current only changes from 0.74 to 0.78 A. Therefore, it can be considered that the electron gun designed in this paper has good sensitivity to voltage.

### Design of uniform magnetic focusing system

Uniform magnetic focusing field is a common permanent magnet focusing system. Due to the distribution of the uniform magnetic field in the whole beam channel, it is very suitable for axisymmetric electron beam. In this part, a uniform magnetic focusing system for maintaining long-distance transmission of double pencil-beam has been proposed. The design scheme of the focusing system is presented by analyzing the magnetic field and the beam envelope generated, and the sensitivity issues are studied. According to the stable transmission theory of single pencil beam^18,19^, the Brillouin magnetic field value can be calculated by Equivalent (2). In this paper, we also use this equivalent to estimate the magnetic field of the transversely distributed double pencil beam. Combined with the electron gun designed in this paper, the calculated magnetic field value is about 4000 Gs. According to the reference^20^, 1.5–2 times of the calculated value is usually selected in actual design.

2 $$\begin{array}{c}{\varvec{B}}_{\varvec{b}}=\frac{830}{{\varvec{r}}} \, \frac{{\varvec{I}}^{\frac{1}{{2}}}}{{\varvec{U}}_{\text{a}}^{\frac{1}{{4}}}}\end{array}$$

**Figure 12:**
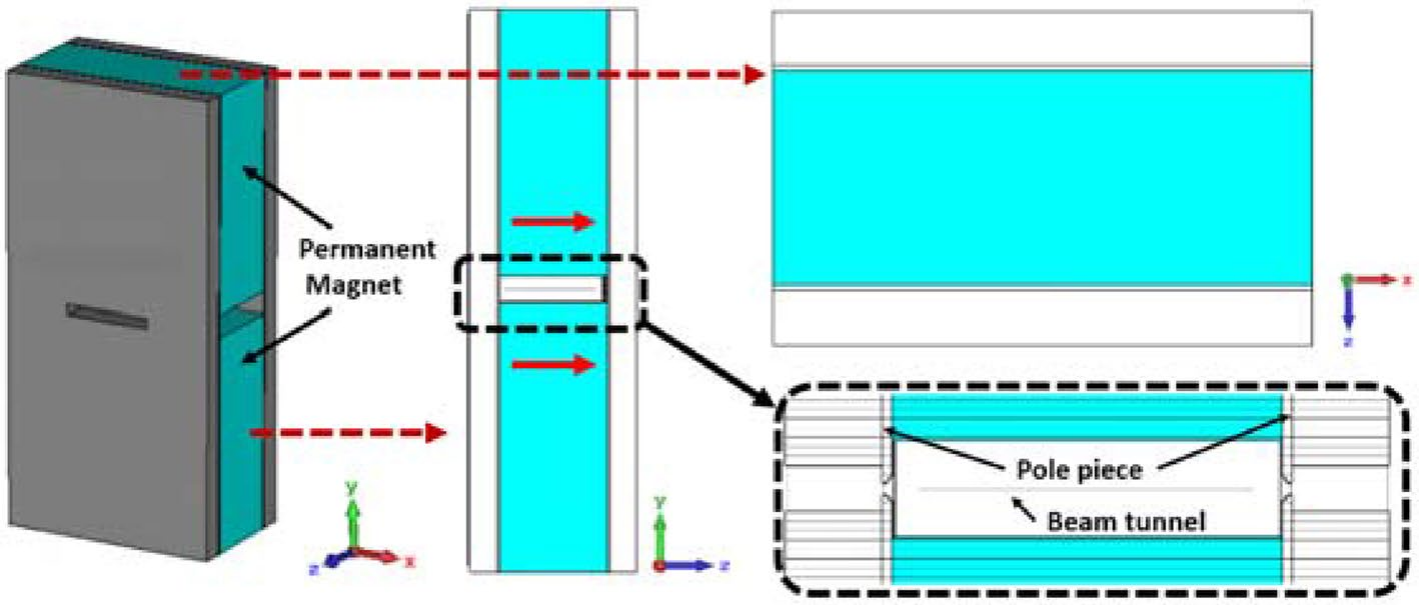
Structure of the uniform magnetic focusing field system.

Figure 12 shows the structure of the uniform magnetic focusing field system. The blue part is the permanent magnet which is magnetized along the axial direction. The material is selected as NdFeB or FeCoNi. The remanence Br set in the simulation model is 1.3 T and the permeability is 1.05. To ensure the stable transmission of the beam in the whole circuit, the length of the magnet is initially set at 70 mm. In addition, the size of the magnet in the x-direction determines whether the transverse magnetic field in the beam channel is uniform, this requires that the size in the x-direction should not be too small^9^. At the same time, considering the cost and the weight of the whole tube, the size of the magnet cannot be too large. So, the magnet is preliminarily set as 150 mm × 150 mm × 70 mm. Meanwhile, in order to ensure that the whole slow wave circuit can be placed in the focusing system, the distance between the magnets is set as 20 mm.

**Figure 13:**
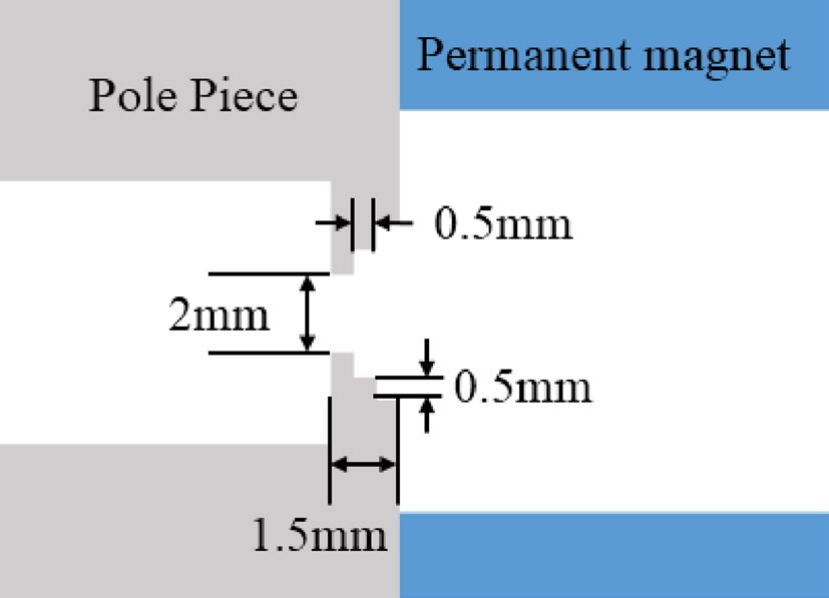
The structure of the pole piece with the stepped hole.

In 2015, Purna Chandra Panda^21^ proposed a pole piece with a new stepped hole in the uniform magnetic focusing system, which can further reduce the magnitude of flux leakage toward the cathode and the transverse magnetic field generated at the pole piece hole. In this paper, we add the step structure to the pole piece in the focusing system. The thickness of the pole piece is initially set as 1.5 mm, both height and width of the three steps are 0.5 mm, and the distance between the pole piece holes is 2 mm, as shown in Fig. 13.

**Figure 14:**
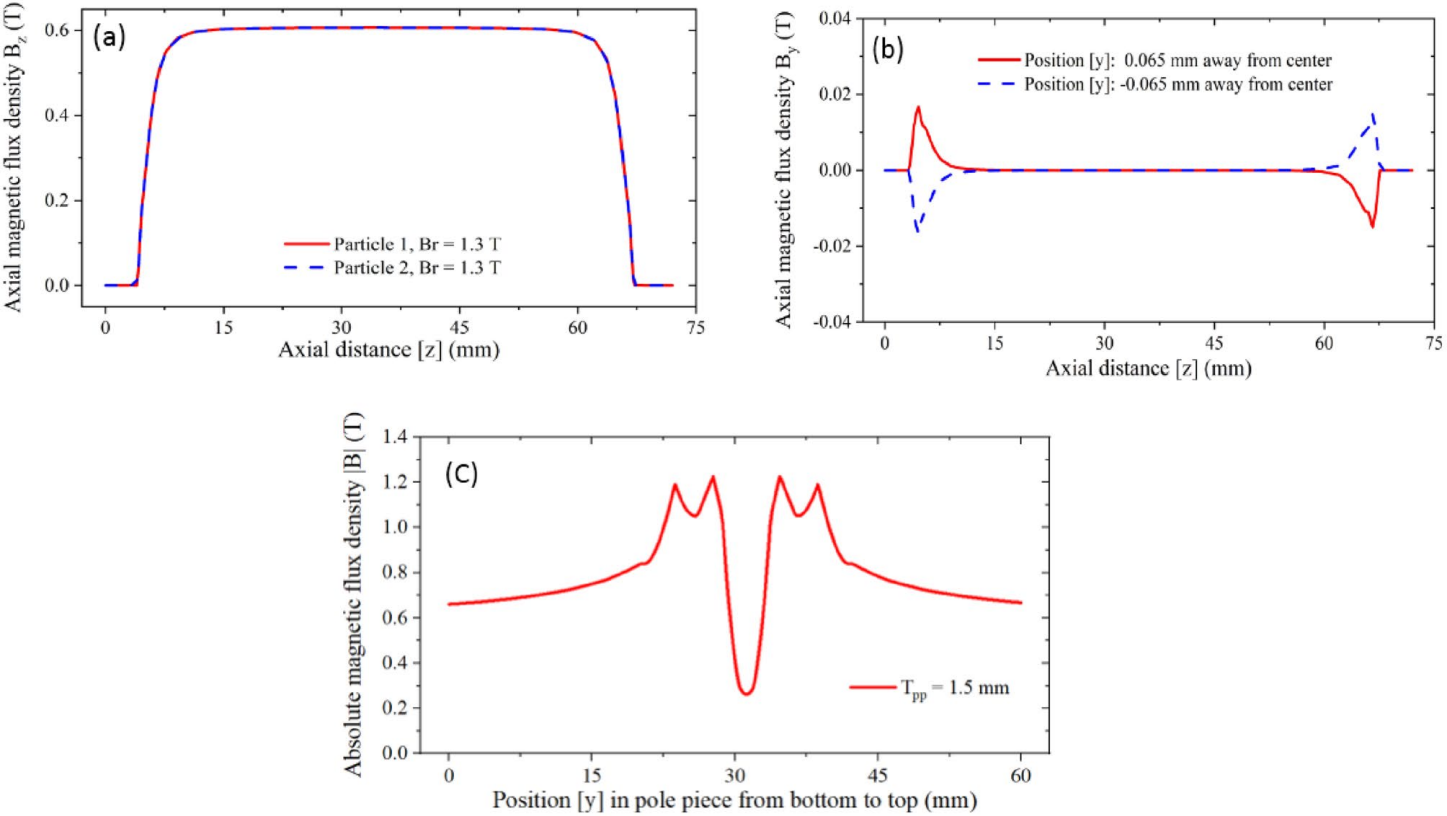
Distribution of the magnetic field intensity with Br = 1.3 T. (**a**) Axial field distribution. (**b**) Transverse field distribution By in the z-direction. (**c**) Absolute values of the field distribution inside the pole piece.

Figure 14a shows the axial magnetic field distribution along the center line of the two electron beams. It can be seen that the magnetic field force along the two electron beams is equal. The value of the magnetic field is about 6000 Gs, which is 1.5 times of the theoretical Brillouin magnetic field to increase the transmission and focusing performance. At the same time, the magnetic field at the cathode is almost 0, which indicates that the pole piece has a good effect on preventing the magnitude of flux leakage. Figure 14b shows the transverse magnetic field distribution ***By*** in the z-direction at the upper edge of the two electron beams. It can be seen that the transverse magnetic field is less than 200 Gs only at the pole piece hole, while in the slow-wave circuit, the transverse magnetic field is almost 0, this result demonstrates the influence of the transverse magnetic field on electron beams can be ignored. In order to prevent the magnetic saturation of the pole piece, it is necessary to study the magnetic field intensity inside the pole piece. Figure 14c shows the absolute value of the magnetic field distribution inside the pole piece. It can be seen that the absolute value of magnetic field intensity is less than 1.2 T, indicating that no magnetic saturation will happen in the pole piece.

### Matching the double-beam gun with the focusing system

**Figure 15:**
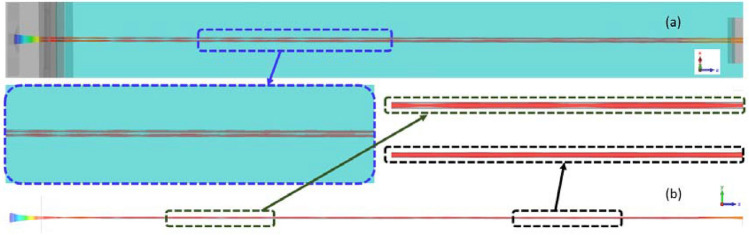
Trajectory of the double pencil beams under the uniform magnetic system with Br = 1.4 T. (**a**) x-o-z plane. (**b**) y-o-z plane.

Based on the CST PS module, the axial relative position of the double-beam gun and the focusing system has been optimized. According to reference^9^ and simulations, the best position is where the anode piece overlaps with the pole piece away from the magnet. However, it is found that if the remanence is set as 1.3 T, the transmission rate of the electron beam cannot be up to 99%. By increasing the remanence to 1.4 T, the focusing magnetic field will increase to 6500 Gs. The beam trajectory on x-o-z and y-o-z planes is shown in Fig. 15. It can be seen that the transmission of the beam is good, the fluctuation is small, and the transmission distance is more than 45 mm.

**Figure 16:**
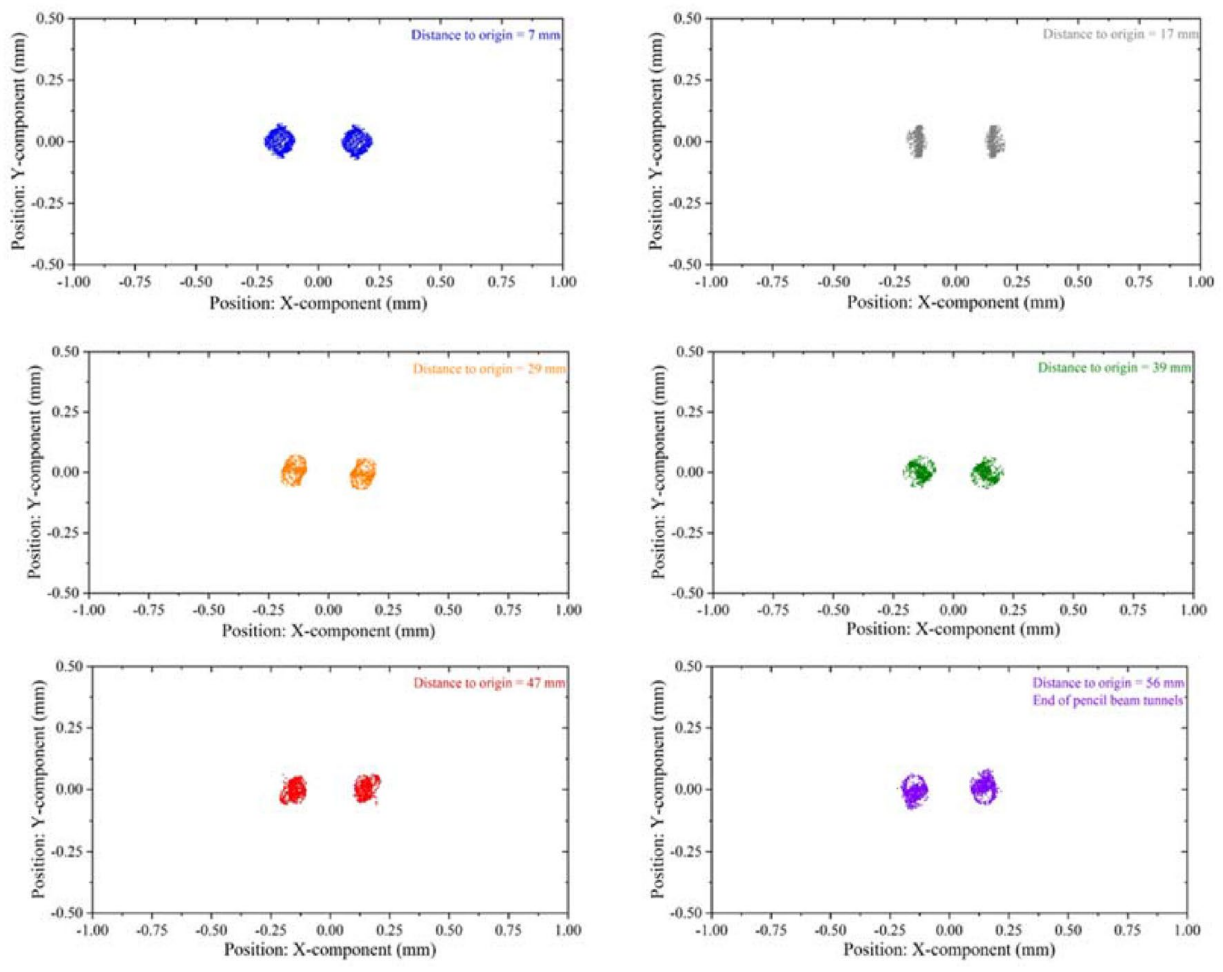
Cross sections of the electron beam at different positions away from the cathode (with focusing system).

**Figure 17:**
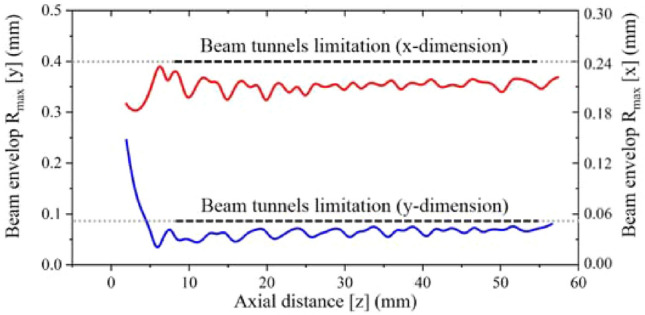
Envelop of the double pencil beams under the uniform magnetic focusing system.

**Figure 18:**
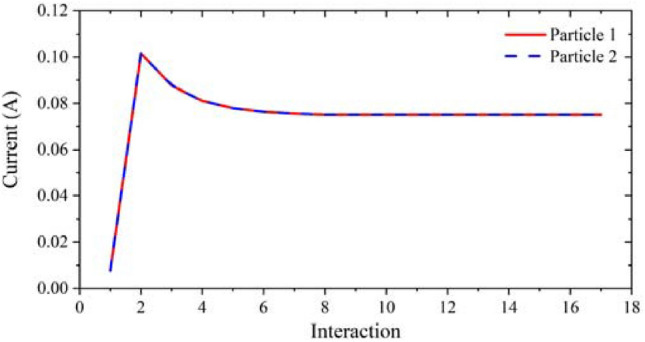
Simulation of the beam current under the uniform magnetic focusing system.

Figure 16 shows the cross sections of the beam at different positions away from the cathode. It can be seen that the shape of the beam section is maintained well in the focusing system, and the diameter of the cross section does not change much. Figure 17 shows the beam envelope in the x direction and the y direction respectively. It can be seen that the fluctuations of the beam in the two directions are very small. Figure 18 shows the simulation results of beam current. Results demonstrate that the current is about 2 × 80 mA, which is consistent with the calculated value in the design of electron gun.

**Figure 19:**
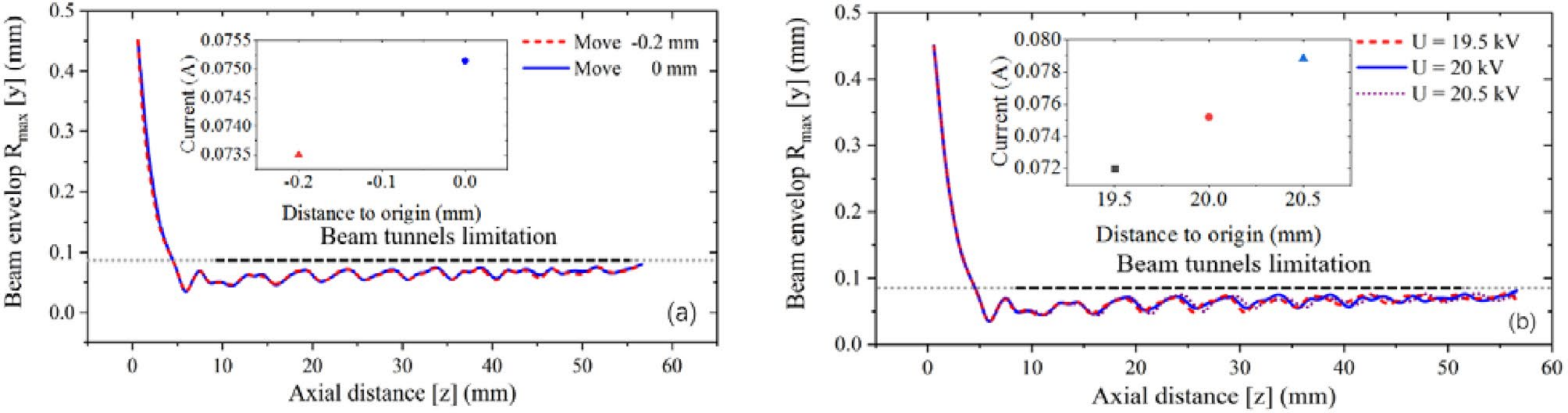
Beam envelope and current result under the uniform magnetic focusing system. (**a**) With the assembly error of 0.2 mm. (**b**) With the driven voltage fluctuation of ± 0.5 kV.

**Figure 20:**
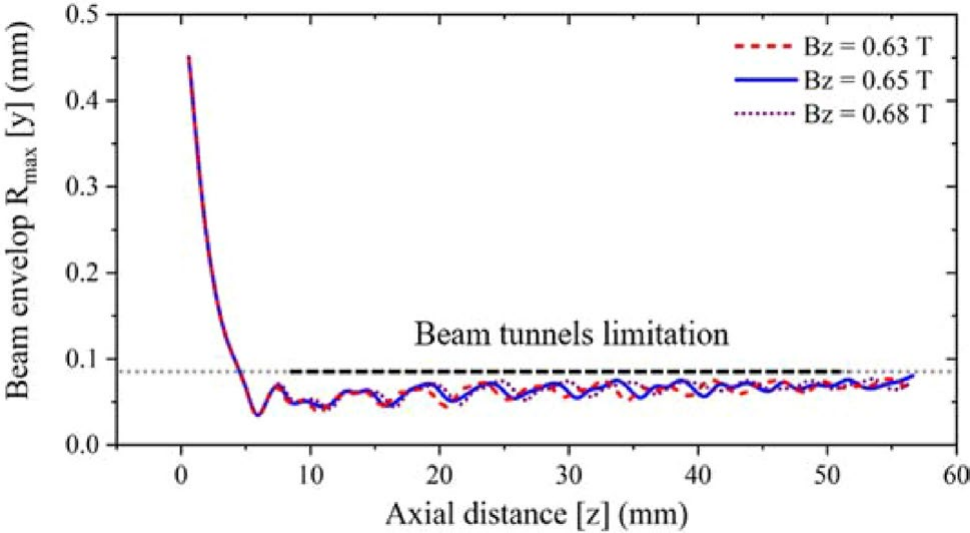
Beam envelope under the uniform magnetic focusing system with the axial magnetic field intensity fluctuation from 0.63 to 0.68 T.

Considering a series of problems such as assembly error, voltage fluctuation and the change of magnetic field intensity in practical processing and application, it is necessary to analyze the sensitivity of the focusing system. Because there is a gap between the anode piece and the pole piece in the actual processing, this gap needs to be set in the simulation. The value of the gap is set as 0.2 mm, and Fig. 19a shows the beam envelope in the y-direction and the beam current. This result shows the change of beam envelope is not obvious, and the beam current is almost unchanged. Therefore, the system is not sensitive to assembly errors. For the fluctuation of the driving voltage, the error range is set to ± 0.5 kV. Figure 19b shows the comparison results. It can be seen that the voltage change has little influence on the beam envelope. For the change of magnetic field intensity, the error range is set from − 0.02 to + 0.03 T. The comparison results are shown in Fig. 20. It can be seen that the beam envelope is almost unchanged, which means that the whole EOS is insensitive to the change of magnetic field intensity.

**Figure 21:**
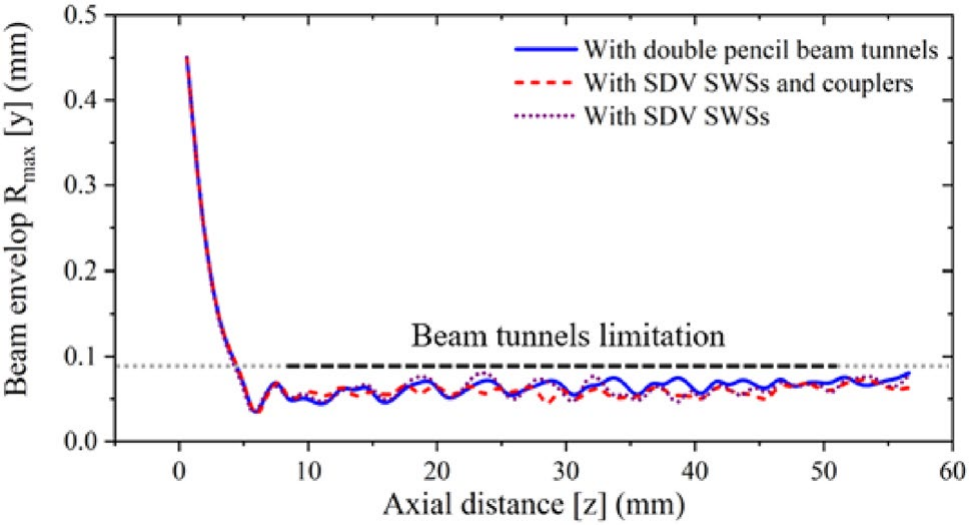
Simulation of the beam current under the uniform magnetic focusing system.

To ensure that the focusing system designed in this paper can match with the HFS, it is necessary to study the focusing system and the HFS together. Figure 21 shows the comparison of the beam envelope with and without loading the HFS. The results show that the beam envelope does not change greatly when the whole HFS is loaded. So, the focusing system is suitable for the HFS of the traveling wave tube designed above.

## Beam-wave interaction with PIC simulation

To verify the correctness of the EOS proposed in section III and investigate the performance of 220 GHz SDV-TWT, 3D-PIC simulation of the beam-wave interaction has been carried out. Because of the limitation of the simulation software, we cannot add the whole EOS into the HFS. Therefore, the electron gun was replaced by the equivalent emitting surface, the diameter is 0.13 mm and the distance between these two surfaces is 0.31 mm, which are the same as the parameters of the electron gun designed above. Due to the insensitivity and good stability of EOS, the driving voltage can be appropriately optimized to achieve the best output power in PIC simulation. Simulation results show that under the driven voltage of 20.6 kV, beam current of 2 × 80 mA (603 A/cm^2^), and the input power of 0.05 W, saturated output power and gain can be obtained.

**Figure 22:**
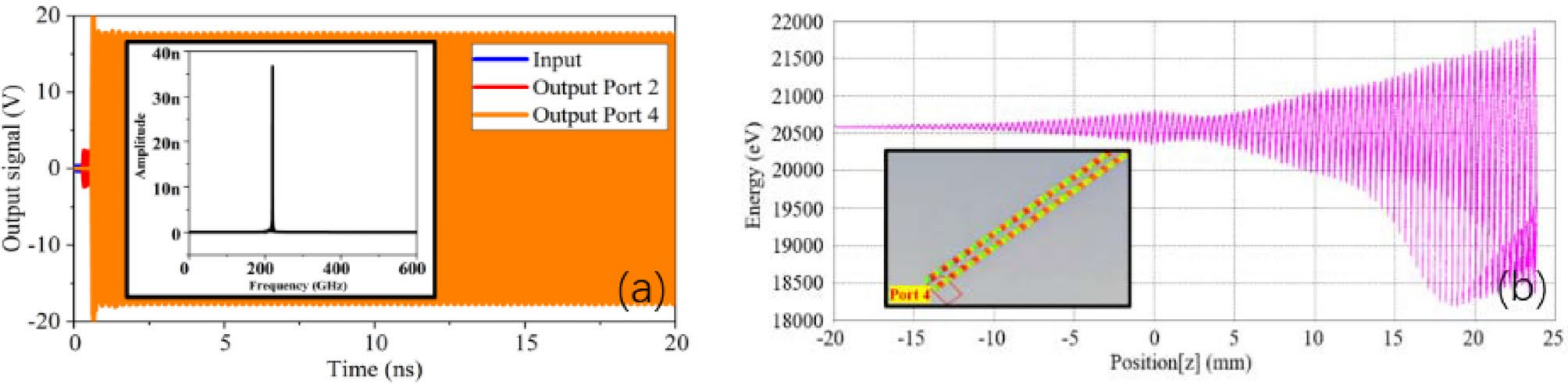
Output signal of SDV-SWS at 220 GHz. (**a**) Output power with frequency spectrum inside. (**b**) Energy distribution of electrons with the electron bunching at the end of the SWS inset.

In order to obtain the best output signal, the number of periods is also needed to be optimized. The best output power can be obtained when the number of two stages is 42 + 48 periods, as shown in Fig. 22a. The 0.05 W input signal is amplified to 314 W with a gain of 38 dB. The output power spectrum obtained by Fast Fourier Transform (FFT) is pure, with a peak of 220 GHz. Figure 22b shows the electron energy distribution with axial position in SWS, and most of the electrons lose energy. This result indicates that this SDV-SWS can convert the kinetic energy of electrons into RF signal, thus realizing signal amplification.

**Figure 23:**
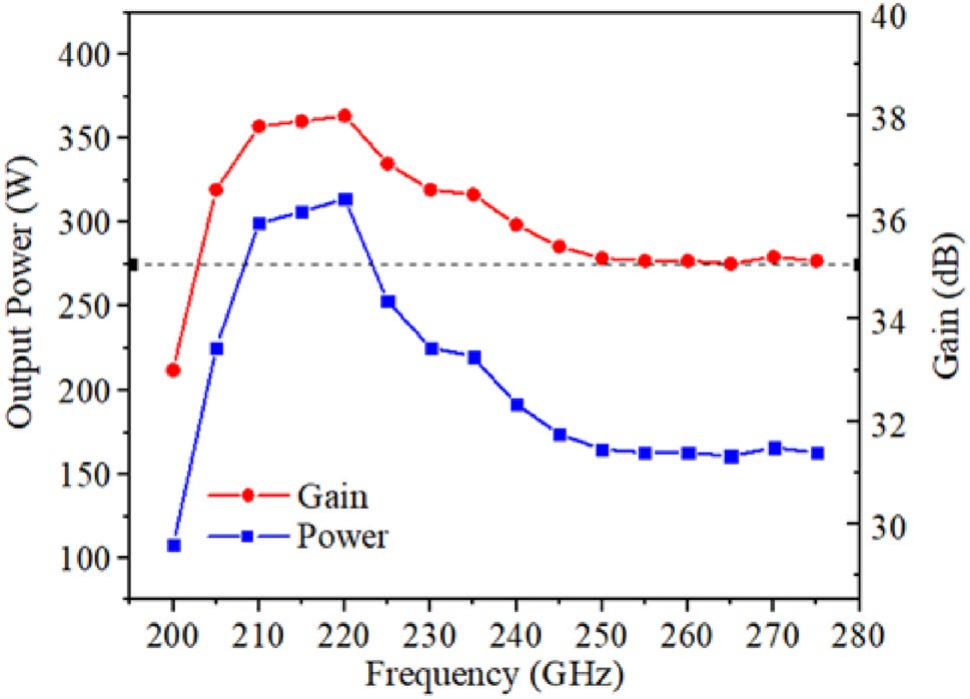
Bandwidth of output power and gain of the whole SDV-TWT.

Figure 23 shows the bandwidth of output power and gain of the double-mode double-beam SDV-TWT. By sweeping the frequency from 200 to 275 GHz and optimizing the driven voltage, the output performance can be further enhanced. This result shows that the 3-dB bandwidth can cover from 205 to 275 GHz, which means the double-mode operation can greatly broaden the working bandwidth.

**Figure 24:**
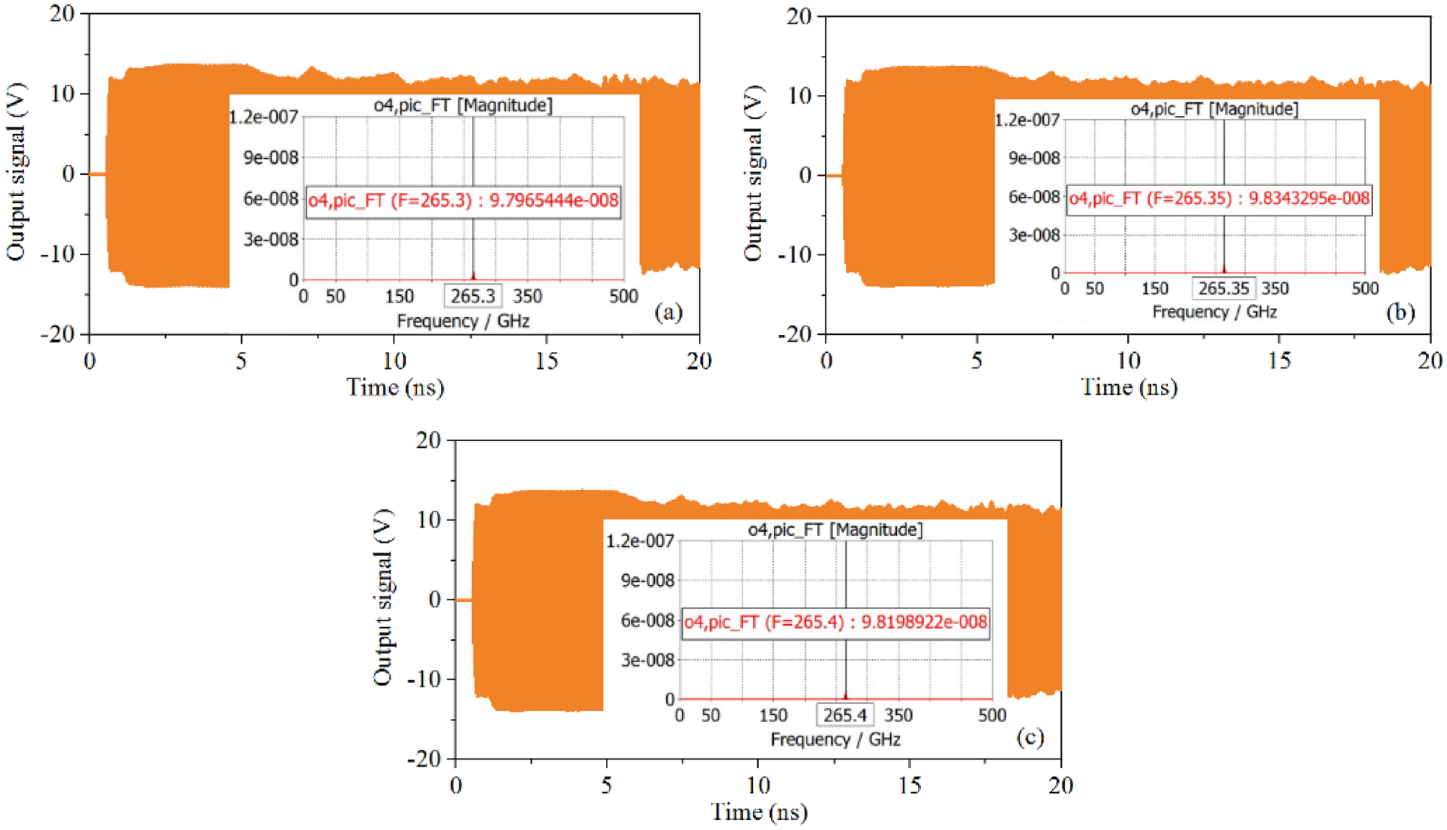
20 ns output signal of SDV-SWS at (**a**) 265.3 GHz, (**b**) 265.35 GHz, (**c**) 265.4 GHz.

However, according to Fig. 2a, we know that there is a stopband between the odd mode and even mode, which may cause the unwanted oscillation. Therefore, working stability around the stop needs to be studied. Figure 24a–c are the 20 ns simulation results at 265.3 GHz, 265.35 GHz and 265.4 GHz, respectively. It can be seen that although there are some fluctuations in the simulation results, the output power is still stable. Frequency spectrum has also been shown in Fig. 24 respectively, and the spectrum is pure. These results demonstrate that there is no self-oscillation near the stopband.

## Fabrication and test for the HFS

**Figure 25:**
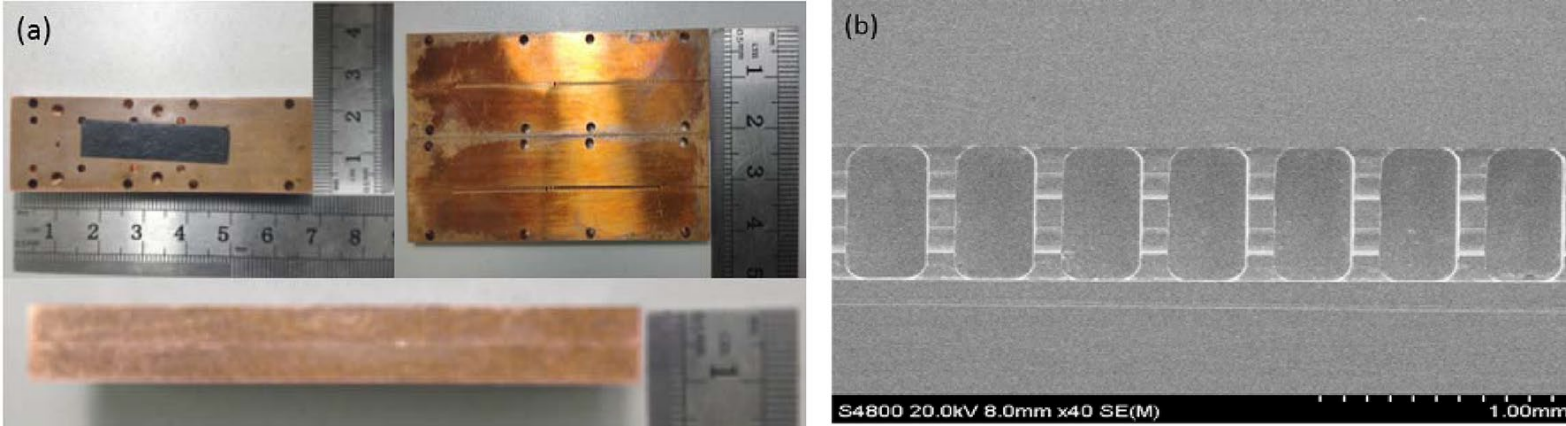
Photos of the fabricating structure. (**a**) Size of the structure. (**b**) SEM images of vanes.

To verify the correctness of the whole HFS, fabrication and measurement are necessary. In this part, HFS has been fabricated by Computer Numerical Control (CNC) technology with a tool diameter of 0.1 mm and a machining accuracy of 10 μm. The material of the high frequency structure was supplied with Oxygen-free High Conductivity (OFHC) copper. Figure 25a shows the fabricating structure. The length of the whole structure is 66.00 mm, the width is 20.00 mm, and the height is 8.66 mm. Eight pin holes are distributed around the structure. Figure 25b shows the structure by scanning electron microscopy (SEM). The vanes of the structure were fabricated uniformly and the surface roughness is good. After precision measurement, the overall machining error is less than 5%, and the surface roughness is about 0.4 μm. The machining structure meets the design and accuracy requirements.

**Figure 26:**
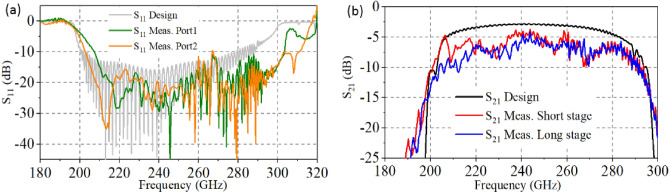
Comparison diagram of the actual test results and simulation of S-parameters. (**a**) S11. (**b**) S21.

Figure 26 shows the actual test results and simulation comparison diagram of transmission performance. Port 1 and port 2 in Fig. 26a correspond to the input and output ports of the HFS respectively, which amount to port 1 and port 4 shown in Fig. 3. The actual measurement results of S11 were slightly better than the simulation results. Meanwhile, the actual measurement results of S21 were slightly worse. The reason might be the excessive material conductivity set in the simulation and the poor surface roughness after actual processing. In general, the actual measurement results fit well with the simulation results, and the transmission bandwidth meets the requirement of 70 GHz, which verifies the feasibility and correctness of the proposed double-mode SDV-TWT. Therefore, combined with the actual fabricating process and test results, the design scheme of ultra-wideband double-beam SDV-TWT proposed in this paper can be used for subsequent fabrication and application.

## Conclusion

In this paper, a detailed design scheme for the planar distribute 220 GHz double-beam SDV-TWT has been presented. Double-mode working operation and double-beam excitation have been used together to further increase the working bandwidth and the output power. Fabrication and cold test have also been carried out to verify the correctness of the whole HFS, and the actual measurement results fit well with the simulation results. For about the designed double-beam EOS, mask portion and control electrode have been used together to generate the double pencil-beam. Under the designed uniform focusing magnetic field, electron beam can transmit long distance stably with good shapes. In the future, the EOS will be fabricated and tested, and the hot test of the whole TWT will also be conducted. This design scheme of the SDV-TWT proposed in this paper fully combines the current mature plane-machining technology, which shows great potential in both performance index and machining assembly. Therefore, according to this paper, the planar structure is most likely to become the development tendency of vacuum electron devices in THz band.

## Data Availability

Most of the original data and analyzed model during this study have been included in this article. More related data can be asked from the corresponding author with the reasonable request.
